# A Novel Method for Precise Onboard Real-Time Orbit Determination with a Standalone GPS Receiver

**DOI:** 10.3390/s151229805

**Published:** 2015-12-04

**Authors:** Fuhong Wang, Xuewen Gong, Jizhang Sang, Xiaohong Zhang

**Affiliations:** 1 School of Geodesy and Geomatics, Wuhan University, Wuhan 430079, China; fhwang@sgg.whu.edu.cn (F.W.); jzhsang@sgg.whu.edu.cn (J.S.); xhzhang@sgg.whu.edu.cn (X.Z.); 2 Collaborative Innovation Center for Geospatial Technology, Wuhan 430079, China

**Keywords:** real-time orbit determination, GPS carrier phase, pseudo-ambiguity, decimeter precision, random walk

## Abstract

Satellite remote sensing systems require accurate, autonomous and real-time orbit determinations (RTOD) for geo-referencing. Onboard Global Positioning System (GPS) has widely been used to undertake such tasks. In this paper, a novel RTOD method achieving decimeter precision using GPS carrier phases, required by China’s HY2A and ZY3 missions, is presented. A key to the algorithm success is the introduction of a new parameter, termed pseudo-ambiguity. This parameter combines the phase ambiguity, the orbit, and clock offset errors of the GPS broadcast ephemeris together to absorb a large part of the combined error. Based on the analysis of the characteristics of the orbit and clock offset errors, the pseudo-ambiguity can be modeled as a random walk, and estimated in an extended Kalman filter. Experiments of processing real data from HY2A and ZY3, simulating onboard operational scenarios of these two missions, are performed using the developed software SATODS. Results have demonstrated that the position and velocity accuracy (3D RMS) of 0.2–0.4 m and 0.2–0.4 mm/s, respectively, are achieved using dual-frequency carrier phases for HY2A, and slightly worse results for ZY3. These results show it is feasible to obtain orbit accuracy at decimeter level of 3–5 dm for position and 0.3–0.5 mm/s for velocity with this RTOD method.

## 1. Introduction

The dynamic changes of Earth environments, and natural disasters, such as earthquakes and forest fires, have been monitored in real-time with spaceborne Earth observation systems (EOS) in the LEO region. The applications of the EOSs require provisions of near real-time position information of LEO satellite platforms with high precision. Therefore, accurate, autonomous and real-time orbit determination (RTOD) using global, abundant, and low-cost GPS measurements onboard LEO satellites has been widely applied to many LEO missions [1,2,3,4,5,6,7,8,9,10,11,12].

The Goddard Space Flight Center (GSFC) and Jet Propulsion Laboratory (JPL) of NASA began the development of algorithms and software about the RTODs using spaceborne GPS data in the 1990s. GSFC developed the GPS Enhanced Orbit Determination Experiment (GEODE) space flight navigation software [2]. The position and velocity accuracy (3D RMS) of GEODE were 7.8 m and 5.9 mm/s, respectively, when applied to TOPEX data collected in the absence of Selective Availability (SA) [3], and the position error (3D RMS) of the orbit results was reduced to 1.0 m after Goldstein’s improvements to GEODE’s algorithm [4]. JPL’s Real-time GIPSY (RTG) software was capable of real-time processing and suitable for embedded systems, such as GPS receivers [5]. Orbit results of 1.5 m for position accuracy were obtained when RTG was used to process SAC-C’s dual-frequency pseudo-range data with the GPS broadcast ephemeris [6]. In addition to the GSFC and JPL researches, the German Aerospace Center (DLR) has committed to the high-precision RTOD since 1999. A miniature single-frequency GPS receiver named “Phoenix-XNS” embedded with an integrated real-time navigation system for orbit determination of LEO satellites was developed by DLR [7]. “Phoenix-XNS” was equipped on X-SAT, a mini-satellite developed by the Satellite Engineering Centre of the Nanyang Technological University at Singapore in 2004 [8], as well as PROBA-2, another mini-satellite developed by the European Space Agency (ESA) in 2010 [9]. Tests demonstrated that the GPS-based navigation system of “Phoenix-XNS” could provide real-time navigation accuracy at 1.0 m level [10].

The onboard RTOD, also known as autonomous orbit determination, has no dependence on ground-based tracking assets, and all of its computations are completed onboard in the embedded system, which usually has very limited computing capacity. The results are required to be delivered within minutes, seconds, or even a fraction of a second after observations are made. GSFC, JPL, and DLR’s algorithm and software seem to use only pseudo-range data. They employ the extend Kalman filter (EKF) to estimate the LEO satellite’s orbit parameters with simplified dynamic models, and the errors of the determined position and velocity are usually at 1.0 m and 1.0 mm/s level, respectively, due to the use of the GPS broadcast ephemeris and large pseudo-range noises [13,14,15]. It appears that the errors of the GPS broadcast orbits and clock offsets were not dealt with in these algorithms.

To improve the accuracy of the onboard spacecraft navigation, considering the requirements of real-time and autonomy at the same time, it is necessary to make full use of the available data, including carrier phases, from the standalone GPS receiver. Although the high-precision carrier phase data of GPS has been widely used in post-processing for precise orbit determination and the accuracy of orbit results has reached centimeter level [16,17,18,19,20,21,22], the post-processing algorithm is not suitable for onboard RTOD. For instance, one is not able to estimate the ambiguity of carrier phase measurements as a constant because only the GPS broadcast ephemeris is available in real-time onboard processing.

HY2A is the first oceanographic satellite of China, and it is dedicated to measuring the wind field, height, and temperature of the sea surface [23]. ZY3 is China’s first civilian mapping satellite, and its central task is to investigate and monitor the land resources in China [24]. For the HY2A and ZY3 satellites, high-precision real-time navigation is essential for their scientific applications, and the real-time orbits at decimeter level or better are required. These two missions are equipped with dual-frequency GPS receivers developed by the China Academy of Space Technology (CAST). The onboard GPS receiver can offer a dual pseudo-range accuracy of 1.5–2.5 m and a dual carrier phase accuracy of 5–6 mm. If only the pseudo-range data is used for the RTOD, it is difficult to obtain real-time navigation accuracy at the decimeter level. Therefore, to obtain higher precise navigation, it is critical to make full use of the other available data, namely the carrier phases.

This paper focuses on the algorithm of the real-time processing of high-precision carrier phase data, especially the mathematical method to deal with the ambiguity parameter of the carrier phases. A new parameter termed “pseudo-ambiguity”, as opposed to the traditional ambiguity parameter, will be defined to combine the phase ambiguity, the orbit, and clock offset errors of the GPS broadcast ephemeris together. A large part of the range error originated from the orbit and clock offset errors of the GPS broadcast ephemeris will be absorbed into the pseudo-ambiguity. By modeling the pseudo-ambiguity as a random walk process and estimating it in a filter process, orbits of higher accuracy are obtained. It will be shown that the introduction of this parameter is a key to achieve real-time orbit results with accuracies at decimeter level for position and sub-mm/s for velocity.

In the following, the dynamical models of LEO satellite orbits and GPS measurement equations will be briefly discussed first. Then, the mathematical definition and properties of the pseudo-ambiguity will be presented in detail, followed by the descriptions of the estimation strategy and SATODS software development. Then, the results of using SATODS to process HY2A and ZY3 data are analyzed. Finally, some conclusions are made.

## 2. RTOD Algorithm and Software

### 2.1. Dynamical Model of LEO Satellite Orbit

The equation of the motion of a LEO satellite can be expressed as:

(1) $$\ddot{\text{r}}=-\frac{GM\text{r}}{r^{3}}+{\text{a}}_{\text{r}}+\text{T}\cdot {\text{a}}_{\text{w}}$$

where $\left(\text{r},\dot{\text{r}},\ddot{\text{r}}\right)$ are the position, velocity, and acceleration vectors of the LEO satellite in a geocentric inertial coordinate frame, respectively, ${\text{a}}_{\text{r}}$ is the total perturbing acceleration, including the non-spherical part of the gravitational attraction of the Earth, lunisolar gravitational perturbations, atmospheric drag, solar radiation pressure, and earth tide. ${\text{a}}_{\text{w}}=\left(a_{R},a_{T},a_{N}\right)$ is the empirical acceleration in radial, tangential, and normal directions, which account for those unmodeled forces, and T is its transition matrix. The empirical accelerations are estimated in the Kalman filter. When the satellite has a complex surface geometry, the interactions of the upper atmosphere and solar radiation with the satellite are difficult to model. In such case, the atmospheric drag coefficient $C_{d}$ and the solar radiation pressure coefficient $C_{r}$ are estimated as scaling factors to account for the complex of the satellite surface. In summary, the parameters in the dynamic models to be estimated are $\left({\text{a}}_{\text{w}},C_{d},C_{r}\right)$.

Various simplifications to the force models in Equation (1) have to be made because of the computation capacity limit of the spaceborne processors. They are well documented in the astrodynamics literature [25]. Detailed settings about the dynamical models will be given in the description of the software development. Considering the accuracy and computational efficiency, a fourth-order Runge-Kutta integrator with a step size of 30 s will be used in the orbit propagation.

### 2.2. GPS Measurements

The dual-frequency GPS receivers onboard the HY2A and ZY3 satellites, developed by CAST, produce pseudo-range and carrier phase data. Two ionosphere-free linear combinations $P_{\alpha }$ and $L_{\alpha }$ of the pseudo-ranges ($C_{1},P_{2}$) and carrier phase measurements ($L_{1},L_{2}$), respectively, can be formed:

(2) $$\{\begin{array}{l} P_{\alpha }=\rho +c\delta_{R}-c\delta^{S}+\varepsilon_{P_{\alpha }}\;\\ L_{\alpha }=\rho +c\delta_{R}-c\delta^{S}+B+\varepsilon_{L_{\alpha }} \end{array}$$

where, ρ is the geometric range between the receiver and GPS satellite, $\delta^{S}$ and $\delta_{R}$ are the GPS satellite and receiver clock offsets, respectively, c is the speed of light in vacuum. $\alpha =f_{1}/f_{2}$ is the ratio of two frequencies. The measurements $P_{\alpha }$ and $L_{\alpha }$ can be expressed by $P_{\alpha }=\left(\alpha^{2}\cdot C_{1}-P_{2}\right)/\left(\alpha^{2}-1\right)$ and $L_{\alpha }=\left(\alpha^{2}\cdot L_{1}-L_{2}\right)/\left(\alpha^{2}-1\right)$, respectively. The parameter B denotes the ambiguity of the ionosphere-free carrier phase measurements. $\varepsilon_{P_{\alpha }}$ and $\varepsilon_{L_{\alpha }}$ are the measurement noises, respectively for $P_{\alpha }$ and $L_{\alpha }$, which include the multipath errors.

### 2.3. Pseudo-Ambiguity Parameter

To use the ionosphere-free phase measurements, the ambiguity B has to be estimated. Although B is a constant in theory, it cannot be fixed if the broadcast ephemeris is used to calculate the orbits and clock offsets of GPS satellites in Equation (2), because the errors of the computed orbits and clock offsets are at the meter level. Without the appropriate processing of the ambiguity, it would be difficult to take full advantage of the high-precision carrier phases.

Therefore, an alternative is proposed to consider the effects of the broadcast ephemeris errors on the orbit determination. Apparently, it is possible to express the geometric range between the receiver and GPS satellite as:

(3) $$\rho =\rho^{*}+d\rho$$

where $\rho^{*}$ is the geometric range between the receiver and GPS satellite, which is calculated using the broadcast ephemeris, and dρ is the range error in the line of sight (LOS) caused by the orbit error of the GPS broadcast ephemeris.

Similarly, the GPS clock offset can be expressed as:

(4) $$\delta^{S}=\delta^{*}+d\delta$$

where $\delta^{*}$ is the clock offset of GPS satellites calculated using the broadcast ephemeris, and dδ is the clock offset error.

Using the above expressions for ρ and $\delta^{S}$, Equation (2) is rewritten as:

(5) $$L_{\alpha }=\left(\rho^{*}+d\rho \right)+c\delta_{R}-c\left(\delta^{*}+d\delta \right)+B+\varepsilon_{L_{\alpha }}$$

Knowing the extreme difficulty to solve the correct ambiguity when only the broadcast ephemeris is available, it would be better to avoid its estimation. A close look of Equation (5) would suggest a new parameter to account for the combined effect of dρ, dδ, and B. In fact, this parameter can be defined as:

(6) A=B+dρ−c⋅dδ

Here, the new parameter A is the termed “pseudo-ambiguity”. B is the true ambiguity. dρ−c⋅dδ is the total LOS error caused by the broadcast ephemeris error. Obviously, the pseudo-ambiguity A is a varying parameter due to the variation of dρ−c⋅dδ.

### 2.4. Properties of Pseudo-Ambiguity

An analysis of the properties of the pseudo-ambiguity would reveal the rational for the definition of the parameter and the approach to estimate it. From the definition, it is only necessary to analyze dρ and c⋅dδ, because B is a constant by its nature. The real data from HY2A is used in the analyses.

**Figure 1:**
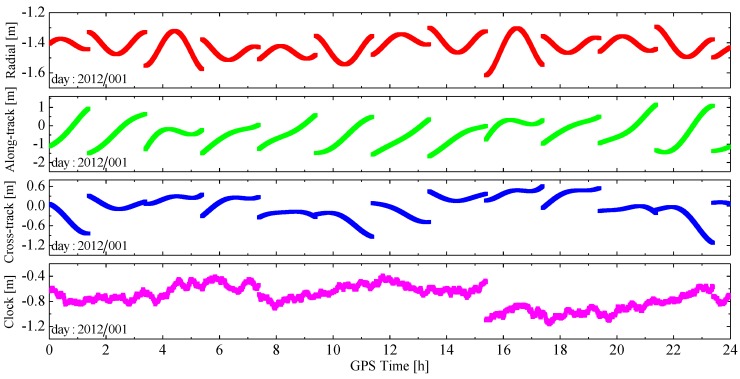
The position and clock offset error of broadcast ephemeris for GPS satellite PRN04.

Firstly, it is noted that the orbit elements and parameters in the broadcast ephemeris are a set of parameters determined through orbit arc fittings, and all the orbits are predicted using the dynamical models by the master station of the GPS system, so the range error dρ caused by the broadcast orbit error should be smooth, and vary slowly. Highly-stable atomic clocks are equipped on GPS satellites and the clock parameters in the broadcast ephemeris are predicted by a quadratic polynomial, so the clock offset error c⋅dδ has a clear trend. These two error characteristics can be seen clearly in Figure 1, which shows the “true” orbit errors in the radial, along- and cross-track directions, and clock offset errors of GPS satellite PRN04. The “true” errors were computed using the International GNSS Service (IGS) precision products as a reference. There are another two important features presented in Figure 1: (1) because the orbit and clock parameters are released every two hours, the error curves are not continuous, which is a result from the use of new ephemeris, and this phenomenon is called “ephemeris switch” in the following text; (2) in addition to the clear changing trends, small random jittering appears in the curve of the clock offset error.

**Figure 2:**
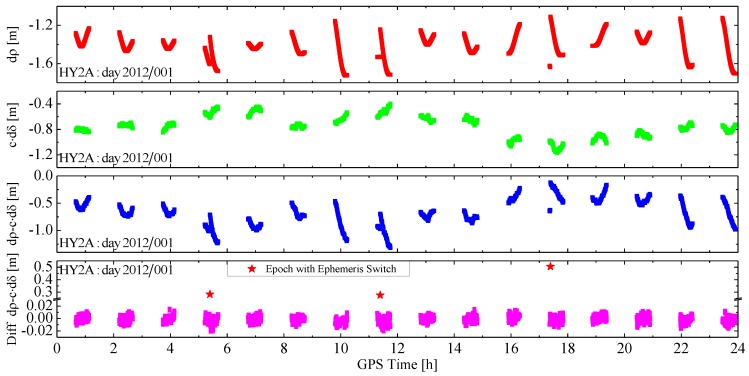
The LOS error and its inter-epoch difference of HY2A and GPS PRN04.

**Figure 3:**
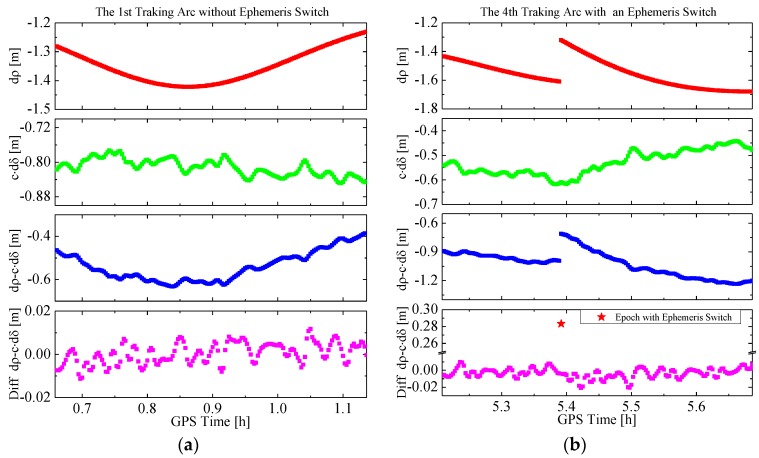
The LOS error and its inter-epoch difference in a tracking arc. (**a**) The first tracking arc of Figure 2 without ephemeris switch; and (**b**) the fourth tracking arc of Figure 2 with an ephemeris switch.

More importantly, it is the combined effect of the GPS orbit and clock offset error of the broadcast ephemeris on the range, the total LOS error dρ−c⋅dδ, between the GPS satellite and the LEO satellite, is of interest. Taking the HY2A satellite and GPS satellite PRN04 on day 2012/001 as an example, Figure 2 shows the range errors caused by the GPS orbit error and the clock offset error, the total LOS error and its inter-epoch difference. Since the trajectory of LEO and GPS satellites are periodic orbits, for every GPS satellite, only part of a GPS orbit arc is visible and tracked by the GPS receivers onboard the LEO satellites. Such a visible arc is called a “tracking arc” in the following text. The thick lines in Figure 2 represent tracking arcs, and the intervals between consecutive lines indicate the GPS satellite is not visible. As can be observed more clearly from Figure 3, if there is no ephemeris switch within a tracking arc, for example, the first arc in Figure 2, the curve of the range error dρ is smooth, and varies slowly, and the curve of the clock offset error dδ presents small random changes, so not only is the total LOS error smooth, and varies slowly, but also it has small random changes up on the smooth curve. However, if there is an ephemeris switch within a tracking arc, for instance, the fourth arc in Figure 2, the curve is discontinuous and the processing of the new curve should be reinitialized. Another important feature is that the inter-epoch differences of the total LOS errors appear as random noises of a normal distribution with its mean very close to zero.

On the basis of the theoretical and practical data analyses above, a random walk process [26] is used to represent the pseudo-ambiguity parameter. The stochastic model of the random walk process for the pseudo-ambiguity can be presented with a difference equation:

(7) $$A_{k+1}=A_{k}+w_{k}$$

where $A_{k}$ is the value of the pseudo-ambiguity parameter at epoch $t_{k}$. Since the inter-epoch difference of the LOS error can be approximated by a normal distribution, as shown in Figure 2 and Figure 3, $w_{k}$ denotes a random variable with Gaussian white noise.

Within a tracking arc, the process noise is set-based on the change rate of the total LOS error. According to Figure 2 and Figure 3, the inter-epoch difference of the LOS error is usually within ±0.03 m for the epoch interval of 30 s, so the change rate is usually less than 1 mm/s. The variance of $w_{k}$ depends on the change rate of LOS error. Of course, if a new broadcast ephemeris is used, or a new GPS satellite is tracked, the pseudo-ambiguity parameter and its process noise should be reinitialized. For the former case, the variance of corresponding pseudo-ambiguity parameter in the estimated state vector should be enlarged appropriately. For the latter case, a new pseudo-ambiguity parameter should be introduced into the filter state and the corresponding variance and covariance should be reset simultaneously.

In summary, a random walk process is proposed to model the pseudo-ambiguity. It can be estimated in the Kalman filtering process. On one hand, the use of the pseudo-ambiguity avoids the need to estimate the carrier phase ambiguity. On the other hand, the estimate of this parameter reduces the effects of the GPS broadcast orbit and clock offset errors on the range; thus, a higher accuracy of the onboard RTOD can be expected. In terms of the algorithm implementation, the number of estimated parameters in the filter remains the same.

### 2.5. Parameter Estimation

An extend Kalman filter is used to estimate the unknown parameters in dynamic models and measurement equations. In total, the filter state

(8) $$\text{X}=\left(\text{r},\dot{\text{r}},{\text{a}}_{\text{w}},C_{d},C_{r},c\delta_{R},c{\dot{\delta }}_{R},A_{1},A_{2},...,A_{n}\right)$$

consists of the spacecraft position and velocity vector $\left(\text{r},\dot{\text{r}}\right)$, the empirical accelerations ${\text{a}}_{w}=\left(a_{R},a_{T},a_{N}\right)$ in the radial, tangential, and normal directions, the coefficients of atmospheric drag and solar radiation pressure $\left(C_{d},C_{r}\right)$, the clock offset and rate $\left(c\delta_{R},c{\dot{\delta }}_{R}\right)$ of the GPS receiver, and the pseudo-ambiguity parameters $\left(A_{1},A_{2},...,A_{n}\right)$ of n-dimension, n is the number of all tracked GPS satellites. Of all parameters, three empirical accelerations are modeled by three first-order Gauss-Markov processes, and the coefficients of atmospheric drag and solar radiation pressure are modeled by two random walk processes. Furthermore, each of the pseudo-ambiguity parameters is represented by a random walk process.

### 2.6. Software

On the basis of the above key technique about the pseudo-ambiguity, a RTOD software SATODS is developed, and it is capable of processing both carrier phases and pseudo ranges. All real data from HY2A and ZY3 missions are processed using SATODS, which simulates the onboard operational scenarios. The model and parameter settings of SATODS take the autonomy, timing, and accuracy requirements of onboard RTOD into account. In order to reduce the computational load, at the same time without loss of orbit accuracy, the dynamical models of LEO satellites are simplified to the maximum extent. The neglected perturbations, including the earth radiation and relativistic effect, are small enough and have no notable effect on decimeter level RTOD. Similarly, simplified precession and nutation models are applied for transformation of coordinate systems. In onboard RTOD applications, the available Earth orientation parameters (EOP) are usually IERS bulletin-A EOP, which may be uploaded irregularly days or months ago. To simulate this scenario, the EOP used in the RTOD computations are obtained from the Bulletin-A files released every seven days by IERS, each of which can provide the predicted EOP for 365 days. In addition, the Dynamic Model Compensation (DMC) algorithm of empirical acceleration from the literature [4] is adopted. The main parameters of DMC algorithm are the correlation time of exponential decay (τ) and the standard deviation of the forcing noise in radial, along-track, and cross-track direction ($\sigma_{R},\sigma_{T},\sigma_{N}$). All the specific model and parameter setting are listed in Table 1.

**Table 1:** Models and Parameters Setting of SATODS.

Model/Parameter	Relevant Setting
Measurement model	
GPS data	Dual-frequency pseudo-range and carrier-phase data, sampling rate of 30 s
GPS orbit and clock	Broadcast ephemeris
Pseudo-ambiguity	Initial standard deviation $\sigma_{0}=5.0\;m$, and the standard deviation of the process noise is $\sigma_{u}=1.0\text{ mm}/\text{s}\times dt$, where dt is the epoch interval.
Dynamic model	
Earth Gravity Field	EGM 2008, adopt 45 × 45 for HY2A and 60 × 60 for ZY3
Luni-solar gravitation	Low precision model, Moon and Sun’s position are computed via analytic method
Earth tides	Low precision model, *k*_20_ solid only
Atmosphere Drag	Modified Harris-Priester model (density), fixed effective area, estimates *C_d_* parameter
Solar radiation pressure	Cannonball model, fixed effective area, estimates *C_r_* parameter
Empirical acceleration	Dynamic Model Compensation (DMC) [4] with a first-order Gauss-Markov model, $\tau \text{=}60\text{ s, }\sigma_{R}:\sigma_{T}:\sigma_{N}=5:40:200\;nm/s^{2}$
Other perturbations	Neglected
Reference frame	
Coordinate system	WGS84
Precession and nutation	IAU1976/IAU 1980 simplified model
Earth rotation parameter	Rapid predicted EOP in IERS Bulletin A

## 3. Flight Data Analyses

### 3.1. Datasets

Experimental datasets covering two five-day intervals, from 2012/001 through 2012/005 and from 2012/032 through 2012/036, respectively, for both the HY2A and ZY3 missions, are processed. Both the ionosphere-free pseudo-range combination $P_{\alpha }$ and the ionosphere-free carrier phase combination $L_{\alpha }$ are used as measurements. A summary of the measurement data sets is listed in Table 2.

**Table 2:** Information on missions and datasets.

Mission	Altitude (km)	Data Arc Year/Days	Noise (m)	
			$P_{\alpha }$	$L_{\alpha }$
HY2A	900	2012/001–005	2.172	≤0.006
ZY3	500	2012/032–036	2.157	≤0.006

In all the RTOD computations, the real-time broadcast ephemeris is used to calculate the GPS orbits and clock offsets. Reference orbits of HY2A and ZY3 for real-time accuracy assessment were generated by the French National Space Research Center (CNES) and Wuhan University (WHU), respectively, using the IGS high-precision GPS orbit and clock products. According to the literature [27,28], the accuracy of the reference orbits is at 2–5 cm.

### 3.2. RTOD Accuracy Analysis

Two solutions were generated from SATODS to examine whether the pseudo-ambiguity is able to reduce the LOS errors. The first solution was from the use of the ionosphere-free pseudo-range combination $P_{\alpha }$ only, and it is abbreviated as “pseudo-range based” for convenient expression. The other solution was obtained by processing both the ionosphere-free pseudo-range and carrier phase combinations, $P_{\alpha }$ and $L_{\alpha }$, and the pseudo-ambiguity was estimated in the filter process. To emphasize the use of the carrier phase data and the effect of the pseudo-ambiguity, this solution is abbreviated as “carrier-phase based”.

**Figure 4:**
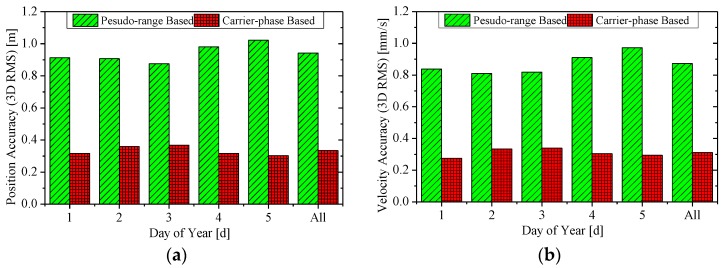
The accuracy (3D RMS) of real-time orbits for the HY2A satellite. (**a**) The position accuracy; and (**b**) the velocity accuracy.

**Figure 5:**
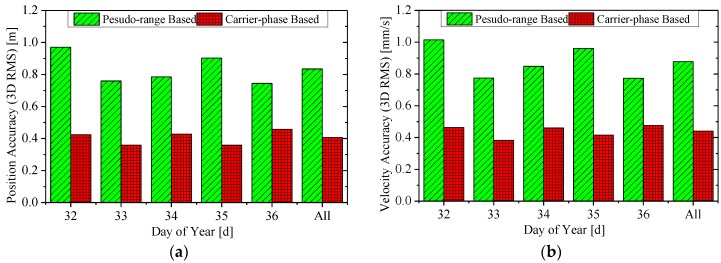
The accuracy (3D RMS) of real-time orbits for ZY3 satellite. (**a**) The position accuracy; and (**b**) the velocity accuracy.

Figure 4 and Figure 5 show the 3D RMS of the RTOD position and velocity errors, where the former is for the HY2A satellite in the interval 2012/001–2012/005, and the latter for the ZY3 satellite in 2012/032–2012/036. The label “All” is for the whole five-day interval. Both the 3D RMS of pseudo-range based and carrier-phase based solutions are presented.

For the HY2A satellite, the position and velocity accuracy of the pseudo-range-based solution are at 0.9–1.1 m and 0.8–1.0 mm/s, respectively, which are similar to GSFC, JPL and DLR’s accuracy level [2,3,4,5,6,7,8,9]. When the ionosphere-free carrier phases are used in the carrier-phase based solution, much better accuracy of 0.2–0.4 m for position and 0.2–0.4 mm/s for velocity, respectively, is achieved.

Slightly worse results are obtained for the ZY3 satellite. A position accuracy of 0.8–1.0 m and velocity accuracy of 0.8–1.0 mm/s are achieved with the pseudo-range based solution. A superior accuracy of 0.3–0.5 m for position and 0.3–0.5 mm/s for velocity, respectively, is realized with the carrier-phase-based solution.

The overall RMS statistics of the real-time orbit accuracy are summarized in Table 3. For the HY2A satellite, the radial (R), along-track (A), cross-track (C), and total (3D) position accuracies of the carrier-phase based solution are better than those of the pseudo-range based solution by 73%, 64%, 64%, and 65%, respectively, and the 3D position accuracy of the carrier-phase based solution is 0.334 m. In particular, the radial accuracy of the HY2A satellite is 8.5 cm, which is better than 0.1 m. Similarly, for the ZY3 satellite, the performance improvements are 47%, 53%, 50%, and 51%, respectively, and the 3D position accuracy of the carrier-phase based solution is 0.407 m.

**Table 3:** The overall statistics of real-time orbit accuracy.

Accuracy (RMS)		HY2A		ZY3	
		Pseudo-Range Based	Carrier-Phase Based	Pseudo-Range Based	Carrier-Phase Based
Position (m)	R	0.312	0.085	0.348	0.186
	A	0.701	0.256	0.714	0.339
	C	0.547	0.197	0.255	0.128
	3D	0.942	0.334	0.835	0.407
Velocity (mm/s)	R	0.624	0.226	0.741	0.360
	A	0.287	0.113	0.357	0.209
	C	0.538	0.181	0.304	0.142
	3D	0.873	0.311	0.877	0.440

**Figure 6:**
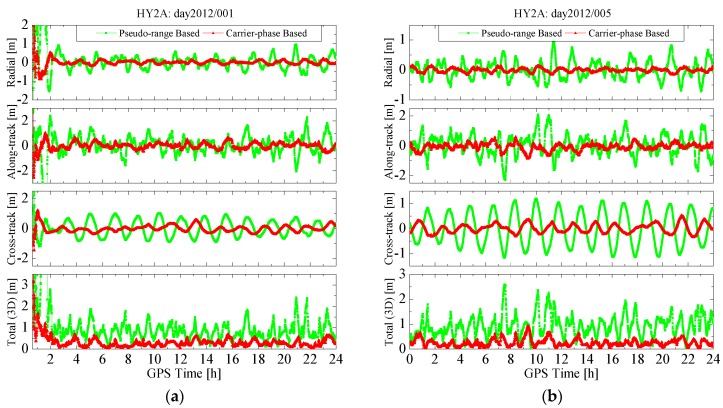
Position errors of real-time orbits for the HY2A satellite. (**a**) Position errors on 2012/001; and (**b**) position errors on 2012/005.

**Figure 7:**
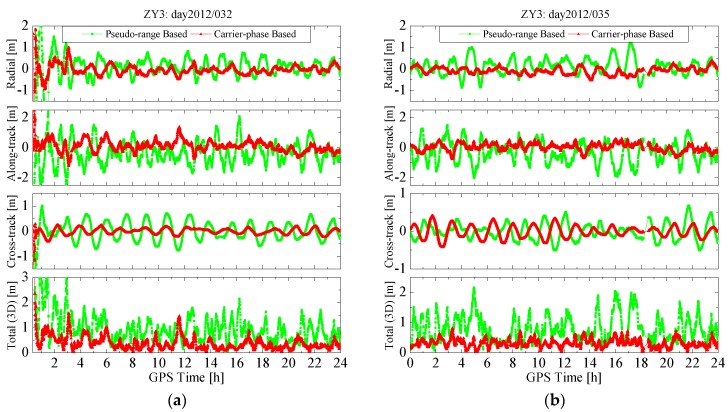
Position errors of real-time orbits for the ZY3 satellite. (**a**) Position errors on 2012/032; and (**b**) position errors on 2012/035.

The position errors of the pseudo-range based and carrier-phase based solutions on several days are shown in Figure 6 and Figure 7, where the former is for the HY2A satellite on 2012/001 and 2012/005, and the latter for the ZY3 satellite on 2012/032 and 2012/035. As can be observed, the error curves for the radial, along-track and cross-track directions, and the 3D position error, for both solutions, present some periodicities. For each solution, the periodicities of orbit error curves are the results of combined effects of many periodic factors, such as the periodicity of the GPS orbit and clock offset error, and the periodical error of dynamic models. However, the variation amplitudes of radial, along-track, cross-track, and 3D position errors of the carrier-phase-based solutions are all notably reduced compared with those of the pseudo-range-based solutions. After the filter process is stabilized, the 3D position errors of the carrier-phase based solutions are all less than 1.0 m, with a RMS value of 0.2–0.5 m. The dynamical models for both solutions are exactly identical, so the results from Figure 6 and Figure 7 demonstrate that the estimated pseudo-ambiguity in the carrier-phase based solution has absorbed a large part of the GPS orbit and clock offset errors and, thus, greatly reduce the orbit determination error.

One should note that, the carrier-phase based solution is more dependent on the quality of GPS carrier-phase data. Some exceptions, such as frequent phase cycle-slips or frequent tracking-losses of GPS satellites, may affect the absorption effect of the pseudo-ambiguity parameter on the GPS broadcast ephemeris error, leading to less significant improvement in the orbit accuracy, when compared with the pseudo-range based solution.

However, the HY2A and ZY3 experimental data sets only cover two five-day intervals, which may lead to an over-optimistic conclusion on the orbit accuracy of the carrier-phase based solution in this study. In order to further demonstrate the orbit accuracy improvement using this novel algorithm, more space-borne GPS data sets from other satellites are tested. This includes two 30-day intervals, from 2010/001 to 2010/030 and from 2013/030 to 2013/060, respectively, for Europe’s MetOp-A [18] and GRACE-A [20] missions. The altitudes of 820 km for MetOp-A and 460 km for GRACE-A are similar to those of China’s HY2A and ZY3, respectively.

**Figure 8:**
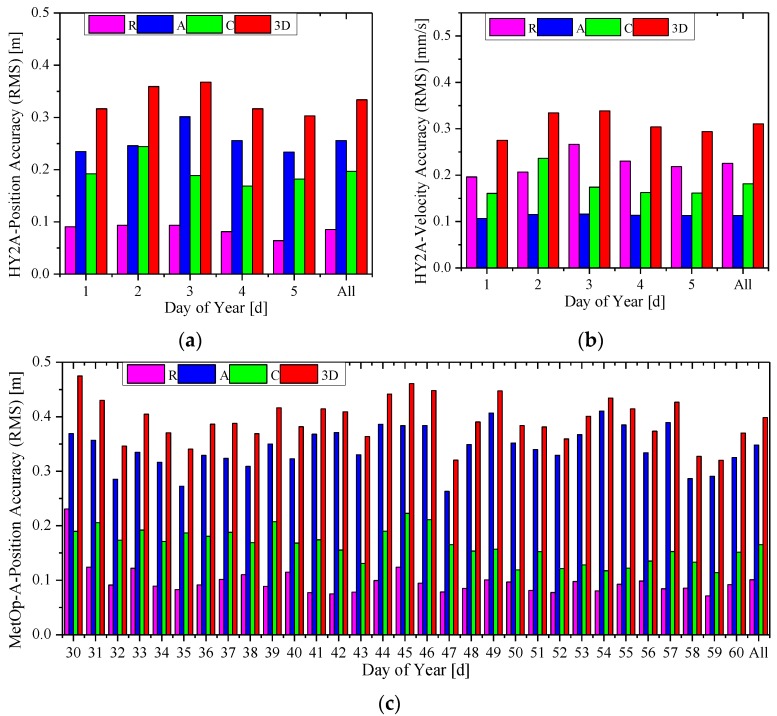

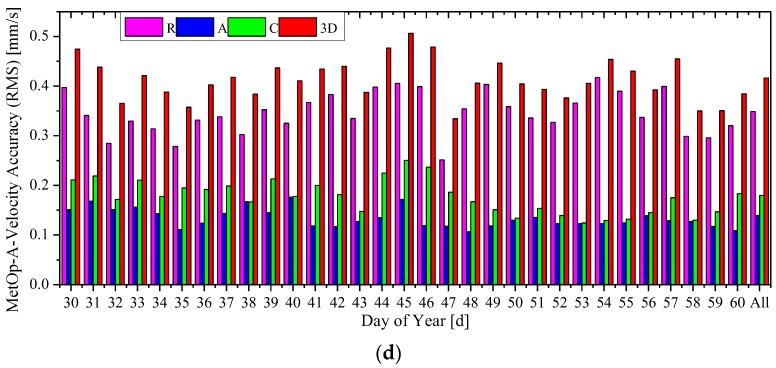
Orbit accuracy of the carrier-phase based solutions for HY2A and MetOp-A. (**a**) Position accuracy of HY2A; (**b**) velocity accuracy of HY2A; (**c**) position accuracy of MetOp-A; and (**d**) velocity accuracy of MetOp-A.

**Figure 9:**
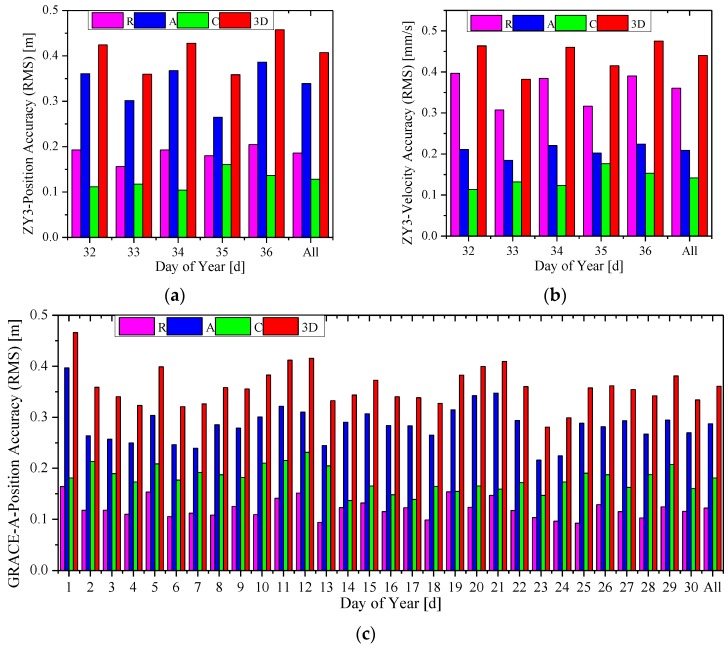

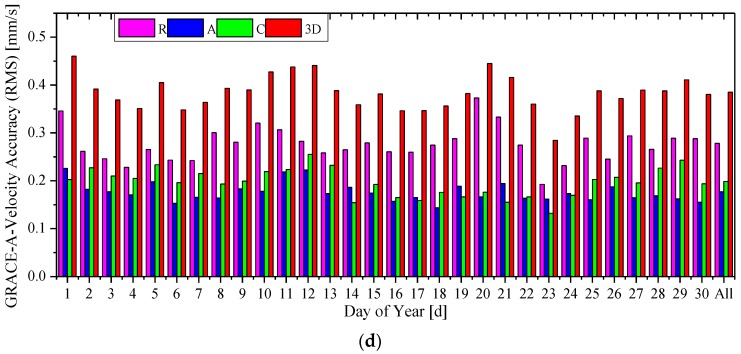
Orbit accuracy of the carrier-phase based solution s for ZY3 and GRACE-A. (**a**) Position accuracy of ZY3; (**b**) velocity accuracy of ZY3; (**c**) position accuracy of GRACE-A; and (**d**) velocity accuracy of GRACE-A.

The daily position and velocity accuracies (RMS) of RTOD results in the radial, along-track and cross-track directions, and 3D position of the carrier-phase-based solution for HY2A and MetOp-A are shown in Figure 8. The accuracies of ZY3 and GRACE-A are shown in Figure 9. The label “All” is for the whole 30-day interval. Comparing subgraph (a) with (c), and (b) with (d) in Figure 8, it can be observed that daily 3D orbit accuracy (RMS) with China’s HY2A satellite is better than 0.4 m, while the daily 3D RMS with MetOp-A is between 0.3–0.5 m. Figure 9 shows that the orbit accuracies of ZY3 are only slightly worse than those of GRACE-A. These two figures demonstrate that the RTOD accuracies with China’s HY2A and ZY3 missions are at the same level with those of MetOp-A and GRACE-A. Although the quality of China’s space-borne GPS receiver is different from that of Europe’s receivers, it is believed that the pseudo-ambiguity parameter plays a major role in achieving accurate carrier-phase based orbit solutions. Therefore, it can be generally concluded that the method presented in this paper can provide the carrier-phase based orbit accuracy of 0.3–0.5 m for position and 0.3–0.5 mm/s for velocity.

### 3.3. Effect of Pseudo-Ambiguity

Orbit results with higher precision are obtained using the carrier-phases compared with those using only the pseudo-ranges. On one hand, this noteworthy performance benefits from the fact that the ionosphere-free dual-frequency carrier phases $L_{\alpha }$ have a ranging noise far lower than that of the ionosphere-free dual-frequency pseudo-ranges $P_{\alpha }$. On the other hand, credits should be attributed to the introduction and estimation of the pseudo-ambiguity, which absorbs a large part of the LOS error caused by the GPS broadcast orbit and clock offset error.

**Figure 10:**
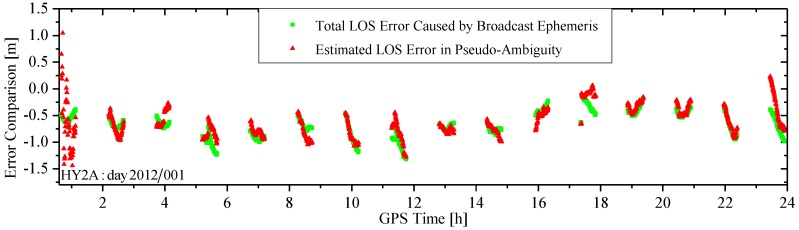
Comparison of LOS errors caused by the broadcast ephemeris and estimated in the pseudo-ambiguity.

**Figure 11:**
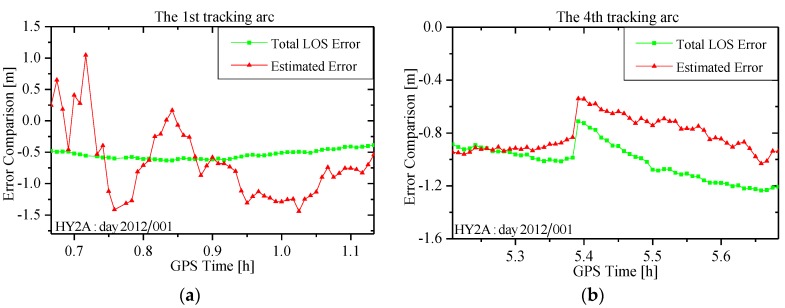
Comparison of LOS errors in a tracking arc. (**a**) The first tracking arc of Figure 8; and (**b**) the fourth tracking arc of Figure 8.

Figure 10 shows two LOS error curves on day 2012/001 between HY2A and GPS satellite. The green curve is the “true” total LOS error curve computed using the GPS broadcast orbit and clock offset, and the IGS high-precision GPS orbit and clock products. The red one is the estimated LOS error in the form of the pseudo-ambiguity (excluding the true ambiguity) using the carrier-phases. To separate the estimated LOS error from the pseudo-ambiguity, the true ambiguity in the pseudo-ambiguity is computed and excluded using the IGS high-precision orbit and clock products via post-processing. As can be observed, the two curves follow a similar pattern, indicating that the LOS error caused by the GPS broadcast orbit and clock offset errors is well absorbed through the estimate of the pseudo-ambiguity. Comparisons of the two LOS error curves in two tracking arcs are presented in Figure 11. In the 1st arc, the two curves do not agree well because the filter process is not stabilized. This means the LOS error is not well absorbed, resulting in less accurate RTOD orbits. Within the fourth tracking arc, although there is an ephemeris switch, the two error curves follow a similar pattern, because the filter process has been stabilized.

**Figure 12:**
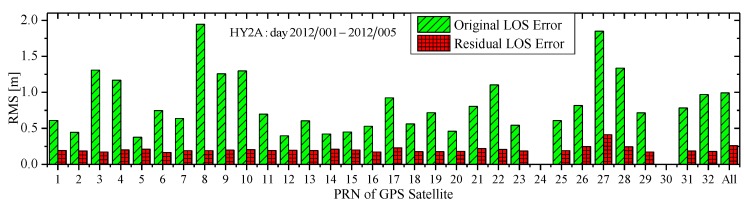
RMS comparison of original and residual LOS error.

Figure 12 shows the RMS of the original total LOS error of HY2A about every GPS satellite in the whole interval 2012/001–2012/005 and the RMS of its residual errors after the pseudo-ambiguity is estimated. The label “All” represents the overall statistics about all tracked GPS satellites. As can be seen clearly, for every GPS satellite, the original LOS error is at 0.5–2.0 m level, but it is reduced to 0.1–0.4 m level due to the absorption effect of the pseudo-ambiguity. Apparently, a large part of the LOS error has been absorbed by the pseudo-ambiguity. In fact, according to overall statistics labeled “All”, the residual error after the estimate of the pseudo-ambiguity is only about 25% of the original LOS errors , which is a primary contributing factor for the realization of the decimeter level (0.3–0.5 m) RTOD.

## 4. Conclusions

Many of the spaceborne Earth observation systems, such as China’s HY2A and ZY3 satellites, require onboard RTOD with decimeter position accuracy. Although these satellites are equipped dual-frequency carrier phase GPS receivers, the limited computing capacity and system autonomy requirement prevent the implementation of complicated orbit determination software, such as the post-processing precise orbit determination software.

This paper presents some of key techniques in developing SATODS software, an onboard RTOD system using dual-frequency carrier phase measurements. Experiment results using tracking data from China’s HY2A and ZY3 missions are presented. One of the key developments is the introduction of the “pseudo-ambiguity” parameter, which is a combination of the phase ambiguity, the orbit and clock offset errors from the GPS broadcast ephemeris. This parameter is modeled as a random walk process, and estimated in the extend Kalman filter. It has been shown to be able to absorb a large part of the LOS error originated from the GPS broadcast ephemeris, which is a key factor to achieve orbit results with accuracies at the decimeter level for position and sub mm/s for velocity. The results demonstrate that the onboard RTOD accuracy of 0.2–0.4 m for position and 0.2–0.4 mm/s for velocity is achieved with the GPS broadcast ephemeris and dual-frequency carrier phase measurements for the HY2A satellite. A slightly inferior accuracy of 0.3–0.5 m for position and 0.3–0.5 mm/s for velocity is obtained for the ZY3 satellite.

The robust results presented here show that the onboard RTOD for LEO space missions can achieve orbit accuracy at 3–5 dm with a standalone dual-frequency GPS receiver and the GPS broadcast ephemeris. Using the algorithm presented in this paper, a better and more accurate onboard RTOD system with higher data utilization has been developed. The system is expected to fly on other missions to enhance real-timeliness of China’s Earth observation systems.
